# Learning needs assessment for multi-stakeholder implementation science training in LMIC settings: findings and recommendations

**DOI:** 10.1186/s43058-021-00238-2

**Published:** 2021-12-04

**Authors:** Mallory Wolfe Turner, Stephanie Bogdewic, Erum Agha, Carrie Blanchard, Rachel Sturke, Audrey Pettifor, Kathryn Salisbury, Andrea Horvath Marques, Marie Lina Excellent, Nitya Rajagopal, Rohit Ramaswamy

**Affiliations:** 1 University of North Carolina, Chapel Hill, Chapel Hill, NC USA; 2 Fogarty International Center, Bethesda, MD USA; 3 Center for Global Mental Health, NIMH, Bethesda, MD USA; 4 Institut pour la Santé, la Population et le Développement, Petion-Ville, Haiti; 5 New Delhi, India; 6 Cincinnati Children’s Hospital Medical Center, Cincinnati, OH USA

**Keywords:** Implementation science, Low- and middle-income countries, Capacity building, Intelligent swarming

## Abstract

**Background:**

Despite significant progress in the field of implementation science (IS), current training programs are inadequate to meet the global need, especially in low-and middle-income countries (LMICs). Even when training opportunities exist, there is a “knowledge-practice gap,” where implementation research findings are not useful to practitioners in a field designed to bridge that gap. This is a critical challenge in LMICs where complex public health issues must be addressed. This paper describes results from a formal assessment of learning needs, priority topics, and delivery methods for LMIC stakeholders.

**Methods:**

We first reviewed a sample of articles published recently in *Implementation Science* to identify IS stakeholders and assigned labels and definitions for groups with similar roles. We then employed a multi-step sampling approach and a random sampling strategy to recruit participants (*n* = 39) for a semi-structured interview that lasted 30–60 min. Stakeholders with inputs critical to developing training curricula were prioritized and selected for interviews. We created memos from audio-recorded interviews and used a deductively created codebook to conduct thematic analysis. We calculated kappa coefficients for each memo and used validation techniques to establish rigor including incorporating feedback from reviewers and member checking.

**Results:**

Participants included program managers, researchers, and physicians working in over 20 countries, primarily LMICs. The majority had over 10 years of implementation experience but fewer than 5 years of IS experience. Three main themes emerged from the data, pertaining to past experience with IS, future IS training needs, and contextual issues. Most respondents (even with formal training) described their IS knowledge as basic or minimal. Preferences for future training were heterogeneous, but findings suggest that curricula must encompass a broader set of competencies than just IS, include mentorship/apprenticeship, and center the LMIC context.

**Conclusion:**

While this work is the first systematic assessment of IS learning needs among LMIC stakeholders, findings reflect existing research in that current training opportunities may not meet the demand, trainings are too narrowly focused to meet the heterogeneous needs of stakeholders, and there is a need for a broader set of competencies that moves beyond only IS. Our research also demonstrates the timely and unique needs of developing appropriately scoped, accessible training and mentorship support within LMIC settings. Therefore, we propose the novel approach of intelligent swarming as a solution to help build IS capacity in LMICs through the lens of sustainability and equity.

**Supplementary Information:**

The online version contains supplementary material available at 10.1186/s43058-021-00238-2.

Contributions to the literature

- This study fills an existing gap in the implementation science literature by systematically assessing implementation science learning needs of stakeholders in low- and middle-income countries (LMIC) and identifying the need for interactive, adaptable, and context-specific training and mentorship opportunities.
- Study findings reinforce other studies in highlighting the need for training opportunities for implementation scientists that address a broad range of competencies in addition to what is considered the standard body of knowledge.
- Based on the diverse set of learning needs expressed by different stakeholders in LMIC settings, this study proposes further research on an innovation called *intelligent swarming* which is a customized approach to capacity building used in the technology support sector.

## Background

As the field of implementation science (IS) grows globally, interest in building researchers’, practitioners’, and policy makers’ capacity to engage in this work worldwide increases. Acknowledging this interest, the journal *Implementation Science* [1] solicited manuscripts describing training and curricula. Over the past few years, several articles have been published detailing training programs in the field, including two by some of the authors of this paper [2, 3]. A recent systematic review by Davis and D’Lima identified 41 capacity building initiatives (CBIs) in eight countries [4].

Despite significant progress, current training programs are inadequate to meet the global need, especially in low-and middle-income countries (LMIC) where many authors have acknowledged the urgent need for IS expertise to address complex, pressing public health issues [5–8]. In their systematic review, Davis and D’Lima pointed out that only 3 of the 41 studies were from relatively low-resource settings [4].

In response, various organizations have implemented training programs targeting LMIC participants in recent years. Examples are the University of North Carolina’s (UNC) partnership with Wits University in South Africa [9], the IS school at the annual conference of the Global Alliance for Communicable Diseases (GACD) [10], funding for capacity building provided by some National Institutes of Health (NIH) Institutes (e.g., the National Institute of Mental Health (NIMH) program on “Research Partnerships for Scaling Up Mental Health Interventions in Low-and Middle-Income Countries,” which requires capacity building activities in countries within the region but outside where the research is taking place), and various training programs sponsored by the World Health Organization’s (WHO) Tropical Disease Research unit [11].

As programs such as these continue to grow, and as the need to train a wide variety of stakeholders expands, it becomes important to understand how well these programs align with the local contexts and needs of learners in LMIC settings. Context has been routinely acknowledged as a factor affecting outcomes in the IS literature [12, 13]. To develop appropriate and useful IS capacity building programs for LMIC settings, a formal assessment of learning needs, priority topics, and delivery methods for different stakeholders in these contexts is necessary. This study attempts to address this gap, drawing on our practical experience with running training programs.

In this paper, we describe the results from such an assessment designed to answer the following research questions:

1. Who are the key stakeholders that need to learn/use IS methods in LMICs?
2. What kind of IS content would be most useful to each stakeholder group, and what is the optimal delivery of that content?
3. What are the implications for future research in IS capacity building in LMIC settings?

Our own impetus for this study was based on unpublished course evaluation data from various IS courses developed and taught by some of this paper’s authors in 2018 and 2020. For example, in 2020, a joint team from UNC and Wits University led a 2-day IS training course in Johannesburg for researchers and practitioners of HIV programs in South Africa. Formal course evaluations revealed significant heterogeneity in the perceptions of the material’s utility and relevance. Researchers felt it was superficial; practitioners found it too theoretical and difficult to apply concepts to their contexts. Moreover, many participants thought the course covered specialized topics when more foundational research skills were needed. Others felt that additional coaching and support were needed to help adapt and translate models, theories, frameworks, and strategies (unpublished observations).

**Fig. 1:**
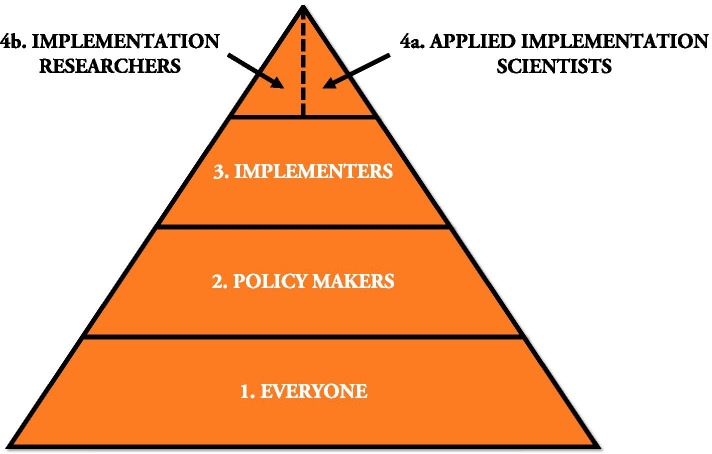
Conceptual “tiered” training model

Based on these findings, we developed a conceptual “tiered” training model (Fig. 1) for IS in LMICs, published elsewhere [14]. This model is based on the premise that instructional systems must educate a large homogeneous population of learners on the basics of a field, with increasingly fewer specialists trained in progressively more complex problems. This model is commonly used in many contexts, such as education, where a general curriculum is offered to all students, with more intensive interventions targeting fewer students with specific needs and interests [15]. Our goal of this research was to assess the suitability of this model to LMIC training in IS settings, develop more precise definitions of the occupants of each of these tiers, create learning objectives for each tier, and identify areas for further research.

## Methods

### Stakeholder groups and definitions

To identify stakeholders for each tier, we first reviewed a sample of articles published in *Implementation Science* in the past 5 years (MWT). We purposively sampled additional articles on dissemination, implementation, and knowledge translation frameworks based on one author’s knowledge of the literature (RR). We included any person or entity the literature identified as having a role in any aspect of the implementation process. We aggregated stakeholders with similar roles and assigned a primary label and definition for each group, along with citations and examples (MWT). We sent the list by email to a purposive sample of twelve individuals known to one author (RR)—each recognized as experts based on their publication record and visibility in IS conferences—and requested them to review the table of stakeholders and identify gaps in the list or edit any definitions. Seven experts supplied extensive inputs, which we incorporated into a final stakeholder table (Table 1) (MWT). Three authors (MWT, RR, and CB) prioritized stakeholders from the list whose inputs would be most critical to develop learning curricula and training programs. The prioritization was based on our first research question, which was to identify stakeholders who could most immediately be potential candidates for IS training programs. These stakeholders (highlighted in Table 1) were those selected for interviews: senior government officials, organizational leaders, implementation researchers, clinical researchers, implementation specialists, staff managers, and implementers.

**Table 1:** Master list of stakeholders involved in implementation of interventions, programs, and policies

Stakeholder	Definition	Alternative names	Example
**Stakeholders providing funding for implementation of programs or interventions**			
Donor	Provides financial support for research, translation, and/or implementation of programs.	Funder	“The **donors’** main role is funding research, KT [knowledge translation] activities, and implementation of research findings [3, 16]. However, Young pointed out that the role of **donors** in KT can be both supportive and disruptive [17]. Failure to address local research priorities and taking control of the research and policy agendas are among the criticisms leveled against **donors** [18, 19]” [20].
Insurer	Pays for services delivered and influences what interventions are allowed and paid for.	Payer, government insurance	“Economic evaluations of interventions usually take a variety of perspectives, including those of the social planner (societal perspective) as well of the entity making the decision whether or not to adopt the intervention being evaluated (i.e., the **payer**). The latter perspective is important because the payer is making the decision of whether or not to adopt the intervention; the reason the societal perspective is also important is because the payer may vary by intervention and disease… Currently, third-party **payers** do not as yet explicitly resource the costs of implementation in their rate-setting decisions, basing reimbursement either on the intervention or, additionally, on the type of provider delivering the intervention” [16].
**Stakeholders responsible for making decisions about implementation policies and priorities**			
Elected official	Has political power and can enact policies to support the implementation of EBPs and/or influence research agendas.	Politician, political leaders	“The roles of **politicians** have been identified as mobilization of communities, dissemination of evidence, and advocacy. However, politicians may face several challenges, including the pressure to respond to their constituencies and political ideological agendas that may influence how they deal with the available evidence [3, 10]” [20].
Policy maker/senior government official	Responsible for formulating policy to support implementation of EBPs but do not have political power.	Civil service employees, regulatory agency staff	“**Policy makers** influence the degree to which research informs policy development, shape the research prioritization process, and impact the actual generation of knowledge [7, 16]. In addition, policy makers play a key role in establishing the required platforms for engagement in KT and in building partnerships between researchers and other stakeholders [21].” [20].
Licensing organizations	Responsible for influencing priorities for EBPs/program implementation.	Accrediting agency staff	“Finally, strategies that attend to the policy context (*n* = 3) can encourage the promotion of clinical innovations through accrediting bodies, licensing boards, and legal systems” [21].
**Stakeholders responsible for allocating organizational resources for implementation of priority policies and practices**			
Organizational executive/leader	Holds a position of organizational authority to make decisions about organizational priorities related to resources and approach to implement EBPs or programs.	Chief executive officer, senior leadership team member, health systems leader	“When management communicates the importance of the implementation of a new practice through its policies, procedures, and reward systems, employees are able to clearly understand that the **leaders** in the organization care about the implementation and use of the innovation, therefore enabling employees to better focus their energy and motivation for that goal. As a result, the overall implementation is more likely to succeed” [22].
Manager	Manages frontline workers in implementation and is supervised by top managers in an organization.	Project managers, nurse managers, team managers	“Studies of **middle managers’** role were conducted in several countries and healthcare settings, across multiple implementation phases, and were related to a range of EBPs; this suggests the breadth of contexts in which middle managers may play an important role in implementing innovations and practice changes” [23]
**Stakeholders contributing to generating implementation-related knowledge or evidence**			
Clinical/public health researcher	Generates evidence for innovative interventions, programs, or policies and can be involved in researching methods for the translation of these innovations into practice.	Investigator, evaluator	“**Researchers** have a key role to play in the synthesis and translation of innovations. Often, the developers of a particular innovation play a major role in its translation. However, it is important to consult or work collaboratively with the intended audience, so that the product developed is more useful to the end user” [24].
Implementation researcher	Generates evidence on factors affecting successful implementation of innovations and researches strategies for addressing these factors.	Investigator, evaluator	“**Implementation researchers** have identified constructs, variables, and strategies that support the sustainable use of evidence to improve outcomes. These constructs and strategies have been synthesized into frameworks and conceptual models that provide a basis for the science of implementation and are used by implementation specialists to support communities to implement, scale, and sustain evidence-informed practices for impact” [17].
**Stakeholders involved in supporting implementation of interventions, policies or programs**			
Implementation support professional	Uses models, frameworks and strategies from implementation research to aid implementers in implementation, scaling, and sustainability of interventions, policies, or programs.	Purveyor, support provider, intermediary, implementation scientist, implementation specialist, facilitator, coach, consultant, technical assistance provider, quality improvement leader	“Implementation researchers have identified constructs, variables, and strategies that support the sustainable use of evidence to improve outcomes. These constructs and strategies have been synthesized into frameworks and conceptual models that provide a basis for the science of implementation and are used by **implementation specialists** to support communities to implement, scale, and sustain evidence-informed practices for impact” [17].
**Stakeholders involved in implementation and local adaptation of interventions, policies, or programs**			
Implementer	Carries out innovations and can contribute to developing local innovations and strategies for effective implementation.	Practitioner, provider, healthcare professional, clinician, staff, implementation team member	“…**practitioners**, who are responsible for program implementation, management, or evaluation, described by Kirchner et al. as the agents who promote evidence-based interventions by using implementation tools and through collaboration with implementation experts in order to employ evidence-based strategies [12]. **Implementers** must systematically and rigorously employ the theory informed frameworks and strategies proposed by the researchers in routine practice in particular settings” [2].
**Stakeholders involved in providing training and education in implementation-related knowledge**			
Implementation science capacity building professionals	Provide implementation science training in academic and non-academic settings.		“Various institutions have begun offering stand-alone implementation science courses to various student audiences [25–26]. For example, the **University of Michigan** Medical School’s Health Infrastructures and Learning Systems program offers, to master’s and doctoral students, a two-semester sequence in ‘Implementation Science in Health’ [25]” [2]. “The intensive workshops offered by the Implementation Research Institute (IRI) and the Training Institute on Dissemination and Implementation Research in Health (TIDIRH) are prime examples in this category [researcher training programs not leading to a university degree, diploma, or certificate]” [2].
**Stakeholders who influence and are influenced by the quality of implementation**			
Patients	Influence implementation research and practice by making decisions about adherence and adoption of interventions, policies, and programs.	Consumers, clients, families, community members	“Considering D&I as a process with multiple phases has implications for how the various topographic levels (i.e., country, system, organization, provider, **patient**) may impact or be impacted by the D&I of evidence-based practices into routine care. Such bidirectional effects are key to conceptual models that recognize recipients of new technologies as not passive but as highly likely to react in various ways depending on characteristics of the context, the innovation to be implemented, and individual differences in health care providers and **patients**” [22].

### Interviewee selection and recruitment

We employed a multi-step sampling approach to recruit semi-structured interview participants, with the goal of approximating a random sample representing each IS stakeholder group. Our approach aimed to ensure the interviewees could demonstrate a connection to the field of IS, were based in or had extensive knowledge of LMIC settings, and were geographically diverse. Two authors (RS, AHM) provided introductions to key members of three global networks: Adolescent HIV IS Alliance (AHISA), GACD, and National Institutes of Mental Health-funded Hubs for Scaling Up Mental Health Interventions in LMICs. We emailed these contacts, with 7- and 14-day reminders, explaining the project and requesting a list of potential interviewees (MWT). The contacts sent back a list of names, or in some cases forwarded the invitation directly to their contacts; one person allowed the research team to post the invitation to a forum of 235 individuals in an online research community.

After assembling the list of potential participants, we individually emailed and sent 7-day reminders explaining the project and linking to a Google form, which we asked stakeholders to complete to indicate their interest in participation (MWT). The form also asked participants to select the IS stakeholder category that best described them. Once respondents filled enough forms for a random sampling strategy to be viable, we emailed one to three randomly selected participants from each stakeholder group to schedule an interview; we sent a reminder in 7 days. We conducted interviews simultaneously with ongoing recruitment using the same sampling process without replacement until reaching the target number of interviewees (five per stakeholder group) (MWT). During interviews, participants mentioned the dominance of English literature in the field. To explore whether there were significant differences in perceptions of learning needs of non-English speakers, we conducted preliminary interviews with one Spanish (MWT) and three French (MLE) speakers, aiming to recommend more detailed research in the future if necessary.

### Data collection

The research team (MWT, RR) developed a semi-structured interview guide (Additional file 1) that we piloted and revised with two IS professionals and a doctoral student at UNC. The phone/video interviews lasted 30–60 min. We obtained verbal consent for and audio recorded each interview. We transcribed and translated French (NR) and Spanish interviews (MWT) verbatim so the analysis team could analyze interviews. For English interviews, the interviewer (MWT) took notes while interviewing, which two team members (MWT, SB) reviewed alongside the recording to clarify and add context to create “memos,” later used to elicit themes. Since most responses were in response to structured questions, this deductive approach to documentation captured the required level of detail while being efficient.

### Data preparation

We used a multi-step process to create a deductive codebook to analyze the memos. We created the initial codebook deductively from the interview guide (MWT). Three authors (MWT, SB, CB) used this version to code one randomly selected interview together to finalize the codebook. Next, to establish intercoder reliability [27], two authors (MWT, SB) coded a randomly selected 25% of the memos individually using NVivo 12 software. We calculated the kappa coefficient for each memo, and the team met to refine the coding process before proceeding to the next memo if the coefficient was less than 0.6—indicating good agreement [28]. Because the kappa values of the first six memos were each greater than 0.8 (indicating excellent agreement), we terminated this step and randomly coded remaining memos independently (MWT, SB).

### Data validation

Validation techniques are recommended to establish trustworthiness of qualitative studies [27]. Techniques include credibility (fit between respondent views and researcher interpretation), transferability (generalizability of results), dependability (research process is logical and clearly documented), and confirmability (conclusions clearly stem from the data) [29, 30]. Our systematic, rigorous approach to coding lent confidence to our dependability.

For confirmability, our data analysis must accurately capture the context of interviewee comments. Therefore, we conducted an internal reflection based on the concept of double loop learning [25]. Double loop learning is intended to address dissonance between tacit assumptions based on mental models that individuals use to make decisions, and the theories individuals articulate as their basis for decision-making. To reduce the influence of our own assumptions and capture the interviewees’ context, two authors (EA, RR) created a rubric adapted from a reflective method called the Critical Moments Technique [31] (Additional file 2). The main interviewer (MWT) used this rubric to identify critical moments from each interview that uniquely described interviewees’ contexts, and to confirm these moments had been included in coding and interpretation.

For credibility and transferability, we sought to guard against implicit bias [32] in data interpretation that could arise from the study team’s location in a HIC. We adapted our validation approach from member checking [33], which involves returning data or results to interviewees to ensure the interview’s intent has been accurately captured. We purposively selected two interviewees and two other individuals who collectively brought a broad range of implementation research, practice, and policy experience from Africa and Asia. We requested that they holistically review our results and the assumptions underlying our interpretation of the data to provide an independent assessment of the contextual credibility of our findings and their generalizability to LMICs. We incorporated feedback from these reviewers to refine our results.

### Data analysis

#### Concordance analysis

For the first research question, we (MWT, RR, CB) performed a concordance analysis between the interviewees’ self-classification of their roles and the interviewer’s classification based on the definitions in Table 1.

#### Thematic analysis

For the second research question, we used thematic analysis [26] to identify patterns from qualitative data. In further elaborations of the method, a distinction is made between “codebook” and “reflexive” approaches to thematic analysis. In the codebook approach, themes are pre-determined by codes derived from interview questions. Themes therefore are both inputs and outputs of the analysis process. In the reflexive approach, coding is open ended, and the themes reflect the analysis output [34]. We primarily followed a codebook approach, though we refined our findings by our internal reflection and external reviewer feedback. This combination of methods assured rigor in the trustworthiness of the data while being sensitive to context. As Braun and Clarke state, “overall, what is important is that researchers use the approach to TA [thematic analysis] that is most appropriate for their research, they use it in a ‘knowing’ way, they aim to be thoughtful in their data collection and analytic processes and practices, and they produce an overall coherent piece of work” ([31], p. 7). Our analysis approach reflects this philosophy.

We followed the Standards for Reporting Qualitative Research checklist [35] (Additional file 3) to guide documentation of methods and results.

## Results

### Interviewee characteristics

Participants worked in over 20 countries spanning Africa, Asia, Latin America, and the Middle East, with most also living in their countries of work (Table 2). A few were currently associated with HICs (e.g., Canada, Japan), but their primary work experience had been in LMICs. Most of the participants who mentioned their prior professional experience had a medical degree or other post-graduate training. Many were responsible for program management or coordination, followed by researchers/academics, physicians, and health financing professionals. The majority had over 10 years of implementation experience but fewer than 5 years of experience in IS.

**Table 2:** Demographics of interview participants (*N* = 39)

	Response	***N***	%
**Countries of work**	South Africa	8	20.5
	Nigeria^a^	5	12.8
	India	3	7.7
	West Africa	3	7.7
	Ghana	2	5.1
	Kenya	2	5.1
	Nepal	2	5.1
	Uganda	2	5.1
	Burkina Faso	2	5.1
	Benin	1	2.6
	Colombia	1	2.6
	Brazil	1	2.6
	Germany^b^	1	2.6
	Japan	1	2.6
	Latin America and Caribbean Region	1	2.6
	Lesotho	1	2.6
	Malawi	1	2.6
	Mozambique	1	2.6
	Canada^a^	1	2.6
	Tanzania	1	2.6
**Past professional experience**	Graduate training^c^ (Master’s degree and/or PhD)	3	15.4
	Medical doctor (with a specific mention of a public health specialization)	6	10.3
	Monitoring & evaluation^c^	4	7.7
	Nurse	2	5.1
	Project management	2	5.1
	Medical doctor (general, no mention of specialization)	2	5.1
	Trainer	1	2.6
**Profession/role**	Program director, manager, or coordinator	18	46.2
	Researcher	4	10.3
	Medical doctor (with a specific focus on public health)	4	10.3
	Professor/Researcher	3	10.3
	Medical doctor and researcher	4	7.7
	PhD Candidate^d^	2	5.1
	Other medical provider^e^	2	5.1
	NGO Executive Director	1	2.6
	Health lead specialist at bank	1	2.6
**Years of implementation experience**	1–5 years	1	2.6
	6–10 years	3	7.7
	11–15 years	6	15.4
	> 15 years	6	15.4
	Did not specify	2	5.1
**Years of IS experience**	< 1 year	3	7.7
	1–5 years	11	28.2
	6–10 years	5	12.8
	11–15 years	1	2.6
	> 15 years	3	7.7
	Did not specify	4	10.3
	No IS training	2	5.1

^a^ One person mentioned working in two countries; respondent is on leave from Nigeria and currently working in Canada. They have been counted in both countries

^b^ Respondent mentioned that they work in Germany, but they have connections to other countries through the project (Tanzania, Uganda, India, Israel, UK)

^c^ One person mentioned past professional experience in monitoring and evaluation, as well as specifically noted a master’s degree; respondent is counted in both categories

^d^ One PhD candidate is also a “health financing expert for the country”

^e^ Includes a general “medical practitioner” response and a midwife

### Concordance analysis results

The concordance analysis explored how interviewees perceived their role within the field of IS and how our team classified them based on the description of their work during the interview. The self-reported stakeholder category and the interviewer assigned category differed among 28% of interviewees (11 of 28). This discordance was because interviewees played multiple roles within their organizations and throughout their careers. The greatest discordance was among two groups. The first was interviewees who classified themselves as clinical researchers or implementers whom we classified as implementation researchers. The reason for this discordance was that we distinguished those who said they focused on developing clinical interventions from those who worked on creating and testing implementation strategies. However, these distinctions were blurred for the interviewees. In addition, some clinical researchers also classified themselves as implementers because they provided services while simultaneously engaged in research. The second group involved those who classified themselves as implementers. We differentiated between those who managed implementation projects (staff managers) and those responsible for the implementation (implementers), but the interviewees did not always make this distinction.

Overall, our interviewees described themselves as “implementers” if they were involved in any aspect of the implementation process. Our interviews revealed heterogeneity in roles, training experience, and stated learning needs that led to more nuanced classifications that can assist in the development of customized and targeted training programs.

### Thematic analysis results

Themes are described in three major categories: experience with IS training, future training needs, and crosscutting contextual issues. The first two themes align directly with interview questions, consistent with the codebook approach to thematic analysis described above. These themes highlight majority perspectives. The third theme arose from our internal reflections and external validation inputs and emphasizes salient learning considerations beyond IS training.

#### Experience with IS training

Table 3 summarizes the perception of IS training interviewees had received to date. About half the stakeholders had no formal IS training; others mostly participated in IS trainings as opportunities arose, for example through workshops or short courses. A few had pursued IS graduate training programs. Modalities through which the interviewees had acquired IS knowledge varied widely, including online resources, online courses, textbooks, self-study, collaborative learning or alliances, and conferences.

**Table 3:** Past IS training of interview participants (*N* = 39)

Code	Response	***N***	%
**Definition of IS**	Putting program/intervention into action by closing research-to-practice gap	19	48.7
	Study and application of scientific methods to design, implement, and scale programs	10	25.6
	Understanding implementation of programs and how they effect change for the purpose of replication/improvement	4	10.3
	Translation of research into policy change	3	7.7
	Improving impact of an intervention	2	5.1
**Application of IS in work**	To frame/guide evaluation of implementation efforts	16	41.0
	To design/adapt new programs or strategies	9	23.1
	To influence policy	6	15.4
	To frame/guide implementation research activities	5	12.8
	To train others to implement	3	7.7
	To guide scaling up	3	7.7
	To help report implementation efforts	2	5.1
	To engage stakeholders	2	5.1
	To guide dissemination efforts	1	2.6
	Ongoing quality improvement/learning	1	2.6
	Unable to describe how IS applies to work	5	12.8
**IS topics and tools used**	Monitoring and Evaluation frameworks	11	28.2
	Other theories of change	6	15.4
	Quality improvement methods	5	12.8
	Determinants frameworks	5	12.8
	Process framework	2	5.1
	EPIS	1	2.6
	Not applying IS-specific framework/tools or unable to identify	15	38.5
**Past IS learning experience**	In-person workshop/short course	14	35.9
	No formal training	16	41.0
	Online resources	7	17.9
	Online courses	6	15.4
	Graduate program	5	12.8
	Textbook/journal	4	10.3
	Self-study	6	15.4
	Alliance/learning collaborative	3	7.7
	Conferences	2	5.1
	Practical experience/application	4	10.3
	Fellowship	1	2.6
	Podcast	1	2.6
**Usefulness of past IS training**	Helped understand what IS is and how it is applied	5	12.8
	Learned new approaches to research	4	10.3
	Provided new ways of framing existing work	3	7.7
	Learned new approaches to job duties	3	7.7
	Basic knowledge	2	5.1
	Usefulness limited by lack of opportunity to apply	2	5.1
	Improved quality of work	2	5.1
	Adequate knowledge	1	2.6
	Non-IS training did not meet IS-specific needs	1	2.6
	Useful to receive training related to project and program management	1	2.6
	Preparation before training and access to materials	1	2.6
**Gaps in training**	How to apply IS (e.g., theories and frameworks) in practice	15	38.5
	How to convey IS to other stakeholders	4	10.3
	Mentorship	2	5.1
	Planning for sustainability	1	2.6
	Importance of exposure to multiple perspectives within IS	1	2.6
	Need for retrospective reflection to determine program impact	1	2.6
	Proposal writing	1	2.6
	Difficulty working with people in other roles	1	2.6
	How to use learnings to influence policy change	1	2.6
	How to display findings (data visualization)	1	2.6
	Evaluating literature and evidence	1	2.6
	How to design research study	1	2.6
**Methods of addressing training gaps**	Consult others	5	12.8
	Self-study	3	7.7
	Mentorship	1	2.6
	Tutoring session at conference	1	2.6
**Barriers to addressing training gaps**	Language	5	12.8
	Time	3	7.7
	Location of training	1	2.6
	Training fees	1	2.6
	Experts are in other countries and busy		2.6
**Ongoing IS learning approach**	Conferences/Workshops	5	12.8
	Online courses	4	10.3
	Through practice	4	10.3
	Self-study	4	10.3
	Reading journal articles	4	10.3
	Meeting with knowledgeable colleagues	3	7.7
	Continuous medical education	1	2.6
	WhatsApp group	1	2.6
	Online databases/resources	1	2.6
**Ongoing learning needs identification**	Through conversations with colleagues in the field	1	2.6
	Through conferences/trainings	1	2.6
	Through reviewing literature	1	2.6
	Through practice	1	2.6

Most interviewees described their training experiences as positive, saying, for example, that trainings helped them understand what IS is and learn new approaches to research or job duties. However, many struggled to define IS. Seventy-five percent of respondents defined IS as (a) closing the research-to-practice gap in implementing programs or interventions or (b) studying/applying scientific methods to design, implement, and scale programs. For example, one interviewee stated:

“Putting research into practice but doing it in an evidence-based manner. You don’t just translate your findings into practice and ask people to just apply it, but you do it in a way that you make sure to monitor the process and evaluate each step, looking at what goes wrong or right and how to incorporate this in a way that can be scaled up.” -Clinical researcher

This definition is consistent with the responses to how IS is primarily applied. Two thirds of interviewees stated that they used IS for evaluation of implementation efforts or designing and adapting new programs. Fifteen percent indicated using IS to influence policy. Overall, even among researchers, the understanding of IS appeared more akin to operational research and process evaluation (e.g., to understand barriers to implementation within a specific program). Only five respondents described using IS to frame or guide implementation research activities, and five were unable to describe how IS applied to their work.

When asked about use of IS theories, models, and frameworks, almost 40% of interviewees reported not using or were not able to identify any IS frameworks or tools. Fewer than ten stakeholders named any IS-specific models, theories, or frameworks. Eleven mentioned using evaluation frameworks, though with the exception of RE-AIM [36], those mentioned were generic, such as theory of change and logic models. This finding reinforces our prior result that there is confusion between IS and process evaluation. Four respondents described CFIR (Consolidated Framework for Implementation Research) [37], one person mentioned EPIS (Exploration, Preparation, Implementation, Sustainment) [38], and two generically described process frameworks.

Even respondents who had undergone formal training in IS mostly described their IS understanding of the field as basic or minimal. The stakeholders named several gaps in training, the most common (40%) being difficulty in applying IS principles to their work and not knowing how to convey IS to other stakeholders. In the words of one interviewee:

“I think there is a gap in understanding how IS can be integrated into each program to enhance the way it works. Very often, there is so much research out of which recommendations emanate, but there is not always guidance on how to implement those recommendations. There is a gap in knowledge of how do you translate those research outcomes into something meaningful on the ground.” -Organizational leader

Most of the other gaps mentioned involved foundational capacity not directly related to IS, such as proposal writing, research designs, or data visualization. Generic barriers to filling these training gaps such as language, time, training locations, training fees, and lack of access to experts were mentioned, but nothing was unique to IS training. In summary, most respondents appeared to view the IS training that they had received as part of general capacity building in program implementation and evaluation rather than as skills in a separate discipline. One interviewee stated:

“In the design of all projects, there is an inclusion of some sort of evaluation of how things are implemented. You include measures of how processes are going out, they use outcomes, outputs, and activities frameworks.” -Implementation specialist

#### Future IS training needs

Table 4 summarizes interviewees’ stated requirements for an ideal IS training program. Reflecting the variation in individual training experience, there was significant heterogeneity in respondent opinions of who should be trained, who should train, how training should be conducted, and the topics that should be covered. However, amidst this variation, some common themes emerged.

**Table 4:** Future IS training preferences of interview participants (*N* = 39)

Question	Themes	Sub-themes	***N*** (%)	Illustrative quotes
**Optimal IS training topics**	Basics of IS	• What IS is and when to use • How IS relates to other disciplines • Models, theories, frameworks • Implementation outcomes, inputs, strategies • Generic presentation so applies to multiple disciplines	14 (35.9)	
	Practical application of IS	• In implementation • Research • Policymaking • Translating research to practice • Scaling community models	10 (25.6)	“So if one could focus on how to implement, rather than how do you develop a policy or strategy or monitor a policy…For instance, if they need to make a shoe…there is all this focus on the shoe must have a lace, a sole…but no concentration on actually making the shoe. How do we implement all these wonderful policies and get from point A to B to C and really do the thing rather than just knowing how to measure it or develop a strategy for it?”
	IS research methods, grant writing, reporting, dissemination	• Disseminate to people on the ground • Experimental design • Practical	10 (25.6)	
	Application of IS in specific contexts (e.g., LMICs)	• Through case studies	6 (15.4)	
	Engaging stakeholders		6 (15.4)	
	Integrating IS into programs/ research (rather than add-on)	• On the ground • Into overall research programs	6 (15.4)	“Because implementation research is not its own topic, we can integrate it into any research we are doing.”
	Optimal data use	• Using data for implementation • How to measure if implementing optimally • Making data-informed decisions	4 (10.3)	“A key issue here is to show results—of how an IS study really provides useful information for you to make decisions.”
	Leadership, behavior change, organizational culture		4 (10.3)	
	How to introduce IS to junior scientists		1 (2.6)	
	Political & economic analysis, stakeholder assessments, cost-effectiveness		1 (2.6)	“I find it difficult to see how IS can in reality be effective without knowledge of health systems. So there is a lot of knowledge in health policy and systems research in the last 20 years which IS can leverage.”
	How to be involved in the world of IS	• At the table in WHO discussions	1 (2.6)	
	Ensuring continuity between funding/partner cycles		1 (2.6)	
	How to access IS information		1 (2.6)	
	Other topics	• Evaluation • Data management, analysis, and visualization • SPSS • Project management • Complexity theory	9 (23.1)	
**Optimal IS training duration**	1 day–2 weeks	• Broken up or together • If in person	12 (30.8)	
	6 weeks–3 months	• 2-3 weeks online, 4 weeks in person, another 1-2 weeks online • Full-time • 3-4 days a week	6 (15.4)	
	3–6 months		1 (2.6)	
	6 months–over a year	• Minimum 1 year if online, or 3-6 months if in person	2 (5.1)	
	Several days over several months	• 6-8 weeks spread out • Short modules with practice in-between • 3-5 day intro with follow up period after applying • With 5- or 1-day intro (3) • If online	15 (38.5)	“…process where people have an opportunity to come back after developing a concept and raising the questions in their own organizations, applying it, and seeing what is achieved through it and how practices change.”
	21 sessions		1 (2.6)	“It’s proven that if you do something more than 20 times, you’re most likely to stick with it.”
	As long as the implementation process		1 (2.6)	“Since it is actually about the implementation of the thing, it needs to be coupled to practical things that people do in their workplace, so it needs to be very minimal lecture time—rather, more practical time on the job.”
	Academic multi-year PhD program		1 (2.6)	
**Optimal IS training format**	Hybrid	• Online first to familiarize participants with content, then in person (3) • In person first, then online (with experts/mentors) (3) • Some instructor contact in person or online for answering questions • Small regional cohorts • Online, in person, then online	25 (64.1)	“COVID will change the way we work. Globally there has been so much virtual linkage, and within [my country], it has propelled us into an online learning environment. A lot of IS input could be online—it’s cheaper, could reach more people, and is as effective…you can still have small groups of people come together face to face as needed where there is geographical proximity”
	Face-to-face	• At the worksite	5 (12.8)	“At the MOH, most people would prefer an online course if it will be a year. But if it were 3-6 months, if I could get an opportunity, I would like it to be face-to-face, if I had resources to support me to go for that training...it would be more interactive.”
	Online	• With real-time support/interaction • Post courses online	6 (15.4)	
**Optimal IS training mode**	Combination of self-study, lecture, workshops, discussion, case studies	• Bring project from own work	12 (30.8)	“Lectures are useful, but in these training contexts, lectures usually end up being people presenting their research, and that isn’t helpful because you can just read about that in their publications.”
	Workshop		12	
	Lecture	• Videos with lecturer and PowerPoint on screen • For introductory material	5 (12.8)	
	Embedded in fieldwork	• With online/telephone mentorship	4 (10.3)	
	Assessment of knowledge		4 (10.3)	“Capacity assessments—assess where people are before so you can tailor the strategy to where people are at…Then you need to evaluate if it works.”
	Self-paced/self-study	• Readings	4 (10.3)	
	Case studies	• Through videos	2 (5.1)	“You need to have a variety of case studies that are context specific, that allow participants to relate to them to understand better. Especially if you’re trying to present a conceptual framework to a case study, then the case study must relate to something that I’ve worked on.”
	Peer learning	• While in-person	1 (2.6)	
	Interactive online discussion		1 (2.6)	
**Optimal IS training participant composition**	Diverse roles	• Including policymakers • Learn from each other • People from different sectors • Everyone who is part of implementation process • Diversifies thinking	25 (64.1)	“Having different stakeholders will make it easier for us to explain our knowledge to other people.”
	Diverse and similar roles		4 (10.3)	
	Similar roles		4 (10.3)	
	Diverse roles but common career level	• Multisectoral	3 (7.7)	“The hierarchical society in [my country] means junior people won't speak openly around senior people.”
	Diverse fields but common implementation issues/ responsibilities		3 (7.7)	“Managers speak the same language. They all complain about people. Policy makers might just be complaining about stakeholders and who didn’t come to a meeting. They won’t relate. It needs to be people facing similar problems in different contexts.”
	Policy makers		2 (5.1)	
	Diverse roles but common level of IS experience		1 (2.6)	
	Diverse roles but same field		1 (2.6)	
	People with research background		1 (2.6)	
**Optimal IS training instructor characteristics/ background**	Interdisciplinary team/multiple instructors	• Experience with research/grant writing (2) • Experience applying IS to policy/practice (3) • Experience using data (1) • At least 2 areas of expertise	23 (59.0)	
	Experience in the field	• Implementing • Guiding policy makers • Working with many stakeholders • High-level implementers or policymakers	16 (41.0)	
	Combination of strong theoretical and practical experience		7 (17.9)	“I would want someone who is academically strong because I would want a strong theoretical base…rather than just a 101 how-to. And experience as an implementer in whatever the discipline and someone who has applied this knowledge, because I would want them to be able to share lessons from the ground.”
	Non-specialist in a particular health area		4 (10.3)	
	Understanding of/experience in context	• LMIC context	4 (10.3)	“I recently went to an IS training…by someone from the UK. There was a bit of discussion after that there was some disconnect between the overly-theoretically driven approach by the lecturer and the actual needs in African contexts. So you need more than an IS researcher from North—you need someone working in the African context as well.”
	Theoretical expertise		4 (10.3)	
	Single coach/ coordinator		2 (5.1)	
	IS research background		2 (5.1)	
	Good teacher	• Lively/fun personality • Good communicators	2 (5.1)	
	High English competency		1 (2.6)	
	Mix of HIC/ LMIC instructors		1 (2.6)	
**Optimal IS post-training support**	Mentorship	• Months or years duration • Monthly or less frequent calls • Face to face or Zoom options • Leading to certification/ability for trainee to coach/mentor (3) • On-demand (2) • Spreading knowledge to others in office • Feedback on application • Proposal writing	25 (64.1)	“One key aspect…which is lacking in many IS programs is mentorship and apprenticeship…In the real world, you can’t just pick up theories and tools. It’s an unfortunate emphasis in the current programs. So much emphasis on the theories and frameworks, but little emphasis on mentorship. Beyond being a science, implementation is also an art. Transferring knowledge within the arts involves lots of learnings which are informal.”
	Network of peers, experts, & instructors; community of practice	• Opportunities to collaborate	10 (25.6)	
	Continuous capacity building	• Monthly seminars where trainees present • CME for implementation • Group activity • Webinar • Booster session	5 (12.8)	
	Small grant		3 (7.7)	“Ideally there would be a small grant attached to that training…That’s when the educational input really becomes useful. Even if that is just offered to a few select participants who have shown promise, that is a quick and easy way to build the practice at the implementation frontline because those people…are going to engage their colleagues around it.”
	Developing a plan after training that is monitored	• Homework to apply models in delivery setting	2 (5.1)	“Maybe during the training, you develop a project that you would go back to implement. Then working with someone—follow up with the trainer to talk about your stage of implementation.”
**Optimal ongoing learning opportunities**	Online library/ resource list		4 (10.3)	
	Mailing list of trainees		1 (2.6)	
	Newsletter or website for updates		1 (2.6)	
**Other/ Miscellaneous**	Need to develop culture of IS	• Everyone should learn IS so can impact population	2 (5.1)	
	Pressure	• Global paradigm shift toward IS necessitates capacity and skills in IS • Under pressure regarding implementation, and time is constrained	2 (5.1)	
	Resource requirements for doing IS well		1 (2.6)	“It's really expensive to do this stuff well…The work itself. Training too, but IS itself is a big undertaking.”
	Umbrella IS organization		1 (2.6)	
	Offer professional training points		1 (2.6)	
	Language barrier of IS materials	• English – French	4 (10.3)	
	Funding mechanism for training?		1 (2.6)	
	Donors affect implementation		1 (2.6)	“Sometimes something that worried me a lot is about monitoring and evaluating IS programs. This is so important, mainly for donor funded programs like PEPFAR and Global Fund things. All of them are worried and concerned about indicators. So they put in place lots of means and resources to ensure they can get indicators. But they aren’t realizing that the presence of these people trying to get information from them about indicators is affecting implementation.”
	Little IS expertise in Africa, but very needed		1 (2.6)	

A majority of respondents emphasized the need for IS topics to cover basic, practical topics. The top six topics that stakeholders felt should be covered were basic IS knowledge [14], practical application of IS [10], application to LMIC contexts [6], engaging stakeholders [6], integrating IS into program planning and evaluation [6], and IS research methods, grant writing, and dissemination [6].

A significant majority of respondents (70%) preferred a combination of online and in-person training. Many interviewees described the need for interactive training programs including elements such as workshops, training embedded in fieldwork, peer learning, and interactive online discussion. There were also suggestions that the training duration should be linked to the distribution of time spent online and face to face. As one interviewee suggested,

“If it’s in person, then a shorter course. In person is much better. If online, then a bit longer. With online courses, not being able to engage that well is a gap.” -Clinical researcher

Twenty-three of 39 (59%) respondents expressed the need for an interdisciplinary team of trainers. An equal number mentioned the need for trainers to have practical experience, with a subset (17%) expressing a preference for trainers who were comfortable with both theory and practice. In addition, some stakeholders specifically stated that instructors should have experience working in LMICs rather than only having training from Western knowledge, and that IS training topics should include application of IS in specific contexts such as LMICs. As one interviewee mentioned,

“I recently went to an implementation science training…by someone from the UK. There was a bit of discussion after that there was some disconnect between the overly-theoretically driven approach by the lecturer and the actual needs in African contexts. So you need more than an implementation science researcher from North—you need someone working in the African context as well.” -Implementation researcher

Sixty-five percent of respondents emphasized the need for training programs to include or be supplemented by mentorship or apprenticeship either during or following training. Other ideas for ongoing support were also mentioned frequently, the most common being communities of practice or learning networks and monthly seminars or other structured events. There was an overall sentiment that training alone cannot build the skills needed to take IS principles from theory to practice. In the words of one respondent,

“Current programs place so much emphasis on the theories and frameworks, but little emphasis on mentorship. Beyond being a science, implementation is also an art. Transferring knowledge within the arts involves lots of learnings which are informal.” -Implementation researcher

#### Crosscutting contextual issues

Several interviewees did not distinguish between implementation research topics and basic research topics. When asked about gaps in their IS knowledge and desire for future training, many stakeholders listed skills and topics related more to general research than to implementation research. Some of the topics suggested were as follows: retrospective reflection to determine program impact, proposal writing, evaluating literature and evidence, designing research studies, data visualization, analysis, evaluation, project management, and use of statistical software.

Similarly, some interviewees stated that applying implementation research was difficult in their countries because basic research capacity was lacking. The ability to conduct implementation research assumes foundational research methods knowledge, and many interviewees described the need to build these skills first or in conjunction with implementation research capacity. In the words of one interviewee:

“Research capacity and output [in my country] is low...so we are just struggling to do basic research—to do operational research. We haven’t been able to move from actually applying research to improving public health. So it is a difficult thing to do IS.” -Implementer

In addition, some interviewees found the emphasis on implementation research training premature when there is still a critical need to develop evidence appropriate to LMICs. Several stakeholders felt that much of the evidence is developed in HICs rather than LMICs, and that interventions are “imported.” For example, many stakeholders worked on projects addressing HIV and/or tuberculosis (TB). The prevalence and impact of HIV and TB in Southern Africa is considerably different than in HICs. As one interviewee stated:

“The evidence is developed in HICs, but LMICs don’t have the baseline data even of the current status and need. I think that first we must generate that evidence, and then we will need to use IS knowledge to scale up.” -Implementer

Interviewees also described how funding structures made conducting and applying IS research a challenge in their countries. In some cases, project funding that came from HICs placed constraints on implementers’ local decision-making authority, optimal measurement, and sustainability of projects. One mentioned that the US-funded project she worked on was “highly prescriptive and mandated by the US,” and two others spoke to the pressure in their US-funded projects to “do things fast,” and measure indicators that impede implementation rather than advance it to targets set by the donor. Another described donors’ impediment to project sustainability:

“So much of the work is donor-driven and therefore finite. At the end of the project cycle, the partner changes…The big development partners like [US funder] have capacity to absorb outputs [referring to IS research], but so much is done by community organizations. How can we involve those partners and capacitate there?” -Organizational leader

Finally, five stakeholders mentioned language as a barrier to learning IS and/or spreading IS knowledge in their countries, pointing out that the vast majority of IS literature is written in English and that, “even the very definition of ‘implementation science’ is purely in English.” A French-speaking interviewee mentioned the lack of IS training materials available in French:

“When I started my master’s in English, I found it extremely challenging to access resources, to read and understand them to differentiate one approach from the other. For the French-speaking world, it’s the fact that training materials and resources in implementation science are unavailable.” -Implementation researcher

Further, the French-speaking interviewees reported that IS is not widely known as a science itself and that some stakeholders involved in IS are not fully aware that they are doing IS work.

## Discussion

### Alignment with existing IS literature

To our knowledge, this is the first systematic assessment of IS learning needs of LMIC stakeholders. Our results reinforce findings from other researchers on training needs and competencies, many of which are not unique to our setting. We will first discuss how our findings reflect training challenges in all contexts and then highlight the unique issues identified by our stakeholders as a rationale for our suggested capacity building approach.

#### Unmet need for IS capacity

Our findings reinforce the enormous demand for IS global capacity and the need to develop approaches to train at scale. Our interviewees reported that they took advantage of every opportunity they could find to be trained. Chambers and Proctor [39], in their summary of the meeting convened by the NIH in 2013 focusing on training in dissemination and implementation (D&I), acknowledged that a broader approach to training is needed than is currently available. Some salient recommendations by the attendees for improving D&I training were to increase training duration, review and update training content, employ train-the-trainer models, and build support networks for training program alumni [39]. The systematic review by Davis and D’Lima reports that many of the current IS training opportunities, such as the NIH-funded training institutes, are extremely competitive and therefore are unable to meet the demand. They also report that conferences and meetings such as the annual NIH conference on the Science of D&I, Society for Implementation Research Collaboration, the Global Implementation Conference, or the Australian Implementation Conference are oversubscribed. Our interviewee sample was composed of those who belonged to various international networks, and they were fortunate enough to have access [4]. There are likely a large number of other researchers and practitioners who are interested but unable to gain access to IS training programs.

#### Targeted training for different stakeholder groups

Our interviewees played a variety of implementation-related roles, emphasizing the need for multiple training programs to meet heterogeneous needs. This finding is aligned with similar observations by other authors. Davis and D’Lima state that many of the capacity building interventions in their systematic review are focused on those who are already experienced researchers, and they identify the need for more novices in the field [4]. Albers, Metz, and Burke propose developing the competencies of “Implementation Support Practitioners” who assist and coach service providers and organizational leaders in implementing evidence-based interventions and practices [40]. Furthermore, Leppin et al. describe the “Teaching for Implementation” framework to train both researchers and practitioners. The interviewees in our study included novices and experts, researchers and practitioners, implementers, and support specialists, who all require thoughtfully designed, targeted training programs [41].

#### Need for a broad set of competencies

Our research has reinforced the need for training curricula that encompass a broader set of competencies than just IS. This finding has also been identified in other research [18, 42, 43]. Metz et al. identified 15 competences for Implementation Support Practitioners across three domains. Applying IS frameworks and strategies is only one of these competencies. Others range from building relationships, to facilitation, to developing teams, to addressing power differentials [18]. In LMIC settings, the WHO collaborated with a consortium of global universities to create a framework of core competencies for implementation research. The framework comprises 59 competencies in 11 domains spanning a broad set of disciplines. Similar to the list of competencies developed by Metz et al., skills in scientific inquiry (e.g., research question formulation, research design, knowledge of IS theories, models, frameworks) that are the focus of IS courses are not central to these competencies. The central component of the WHO training framework is learning how to “identify appropriate stakeholders, engage with them meaningfully, form robust collaborations and implement change via these collaborations throughout the IR process” [19]. Our findings support the need for an IS training model to teach many topics from a broad set of competencies to a wide audience, rather than a specific set of topics taught in depth to a few.

#### Interactive support

A majority of our interviewees acknowledged the critical importance of what Darnell et al. [44] call “interactive resources” such as workshops, conferences, and mentorships and commented on the difficulty of finding suitable mentors. These results are aligned with the evidence illustrating the value of mentoring to foster research collaborations [45] or to facilitate learning on the application of IS concepts, theories, and frameworks [46], as well as with findings from a content analysis of 42 D&I resources that found that mentoring, networking, and technical assistance were the least common forms of available interactive resources [44].

### Application to LMIC settings

The fact that many of our findings related to the learning needs of IS stakeholders in LMICs have also been reported in the literature focusing on high-resource contexts does not imply that the situation across these contexts is identical. While the issues may be similar, interpretation and strategies to address them must be different for our interviewees for two reasons. The first reason is the question of scale. The gap in training capacity relative to the demand, the range and scope of training that is required, and the effort needed to develop a pool of qualified local mentors with knowledge of culture, customs, and language in various geographies is significantly greater in LMIC settings.

The second consideration is structural and has to do with the way in which the participants in our interviews gained access to training. As our concordance analysis showed, our “stakeholder groups” were not stable, homogeneous entities. At various times in their careers, professionals can be researchers, practitioners, policy makers, or organizational leaders, sometimes simultaneously. Stakeholders may identify themselves as belonging to a particular group, even if their roles and activities make them more likely to be classified differently. This is in part because many of our stakeholders build their professional careers by working on a variety of research or program implementation projects dictated by the availability of Western grant funding. Depending on what was needed for the purposes of the project, our interviewees demonstrated an impressive ability to be flexible and play different roles. However, this flexibility comes at a cost because it could be a barrier to achieving sustained expertise.

We found that this mobility across projects also resulted in fragmented access to training opportunities. Many of our interviewees acquired their skills opportunistically, determined by the training that was available to them as a consequence of working on a project. This need to take advantage of whatever training is available results in a patchwork of competencies with some areas of strength and other critical areas in which there are substantial gaps. Moreover, because different individuals attend different training programs, there is no infrastructure for creating the cohesive ongoing learning communities similar to those that are available in the US to Implementation Research Institute (IRI) or Training Institute for D&I Research in Health (TIDIRH) fellows to advance their skills and expertise [45, 47].

Finally, most of our interviewees participated in training opportunities that were led by instructors or used content from high-income settings and were primarily delivered in English. “Context matters” is a core tenet of IS (e.g., [13, 48]), but context-appropriate training is hard to come by. Many interviewees expressed the need for trainers and mentors who had knowledge and experience of local issues and were able to bring local examples to their teaching.

These structural factors reflect the disparities in how funding for programs is generated, where expertise is located, and how leadership is distributed, not just in IS, but in global health in general. Recent writing by Abimbola (2019) addressing power imbalances in authorship [49] and content of global health articles and by Baumann and Cabassa (2020) and Snell-Rood (2021) on the need for IS theories, models, and frameworks to address inequities and power differentials has drawn attention to these issues [50, 51]. While a concentrated focus on these issues is critical, it is important to mention that our interviewees did not frame their responses through the lens of colonialism or power differentials, but presented their learning needs as a pragmatic problem for which solutions are needed. It is in this context that we propose the ideas that follow.

### Imagining new learning models

The heterogeneity in current capability, the breadth of competencies required, the scale at which capacity needs to be built, and the critical need for context-appropriate learning revealed in our interviews suggest that traditional classroom-based training models are likely to be of limited value. Rather, there is the need for what Eboreime and Banke-Thomas call the “art and craft” of implementation training, that emphasizes “relationships, supportive supervision and coaching” [52]. Our findings suggest the need for a learning approach that builds upon individual participants’ existing strengths and knowledge, facilitates generation of context-specific IS knowledge, and is taught in an interactive format, with mentoring as an integral component. Situational learning theory [53] may serve as a useful frame for developing a learning model. Situational theory states that learners progress from novice to expert through engagement in communities of practice where learning opportunities arise through social interactions with others involved in the same pursuit. Rather than primarily investing in classroom training, where an external expert delivers a body of content that may not be relevant or useful, a promising approach might be to create learning networks focused on implementation research, practice, or policy. Facilitator teams with IS knowledge and understanding of the local context would mentor participants in systematic approaches to test, adapt, or create locally relevant implementation frameworks, tools, and strategies. These networks would be different from the global networks such as GACD or AHISA in that they would intentionally focus on a local region or geography, explicitly recruit an interdisciplinary team of advisors who combine technical IS knowledge with a deep understanding of context and promote learning through a variety of relationships (e.g., apprentice/expert, peer-to-peer, mentored groups) that emphasize practice. To our knowledge, these types of networks do not exist today in LMICs.

Wenger defines three necessary characteristics for a community of practice: (1) the *domain—*the shared area of competence that the learners seek to advance; (2) the *community—*the intentional group of learners committed to relationships to further learning; and (3) the *practice—*the collection of activities, processes, and interactions through which learning occurs [54]. Learning networks based on these characteristics may be attractive models when there is agreement about the domain. But our interview results revealed significant variation in background and knowledge even within the domain of IS, and also wide differences in learning priorities. As mentioned earlier, the WHO core competency framework for implementation research in LMICs [19] identifies 11 domains ranging from engaging stakeholders, to conducting ethical research, to research designs—each an area around which a learning network can form. A single model to facilitate learning of IS seems unlikely to meet the diversity of need. Our findings suggest that dynamic models that bring situational learning to the individual level by providing customized, adaptive, and agile learning environments still rooted in mentoring, relationships, and practice are necessary. Rather than establishing a predefined body of knowledge and a rigid instructional structure, the learning process and the learning support would emerge from the scope and complexity of the need.

### Drawing from the service sector: directions for future research

**Fig. 2:**
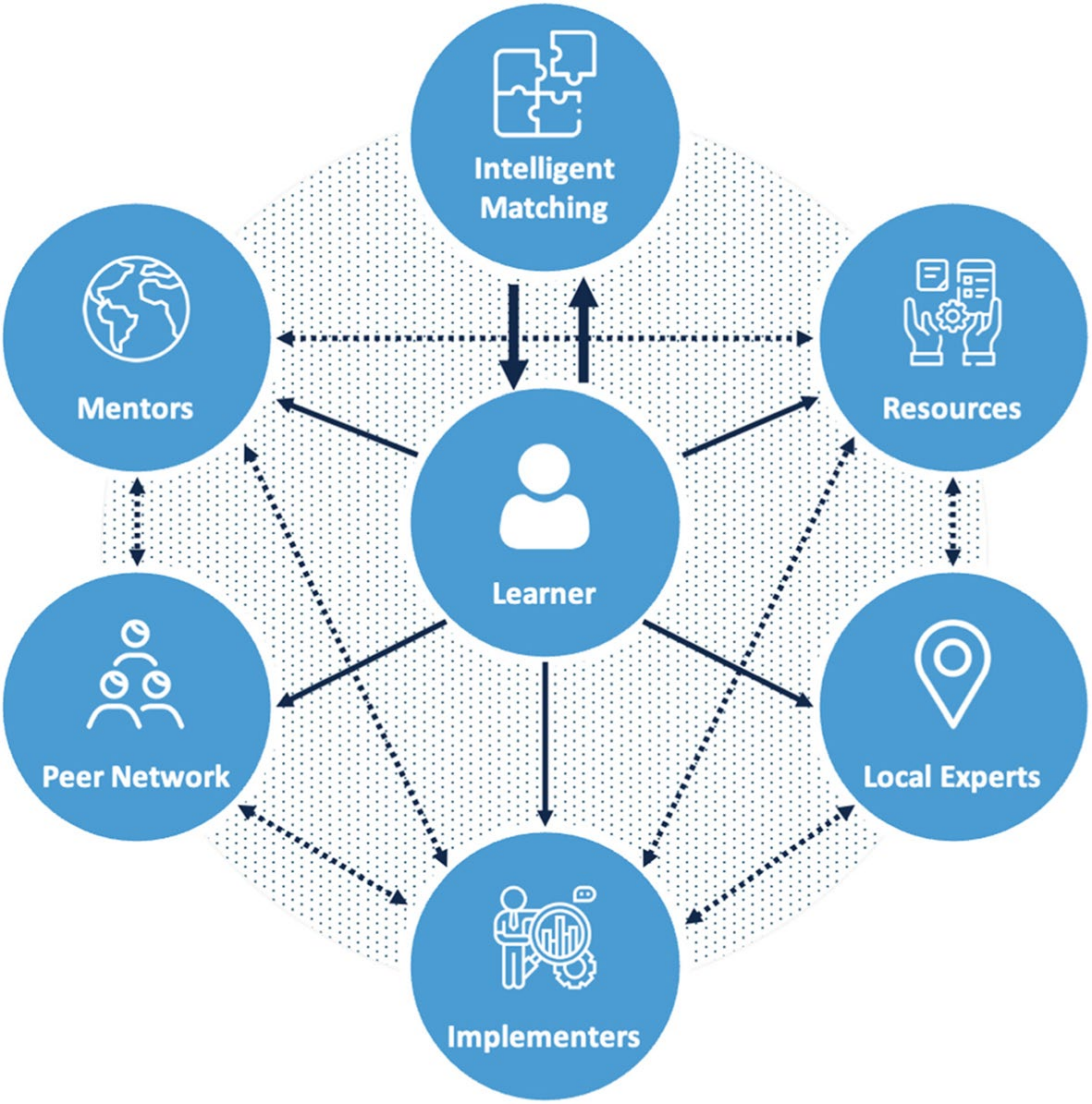
Illustration of an “intelligent swarm” network of support resources

**Fig. 3:**
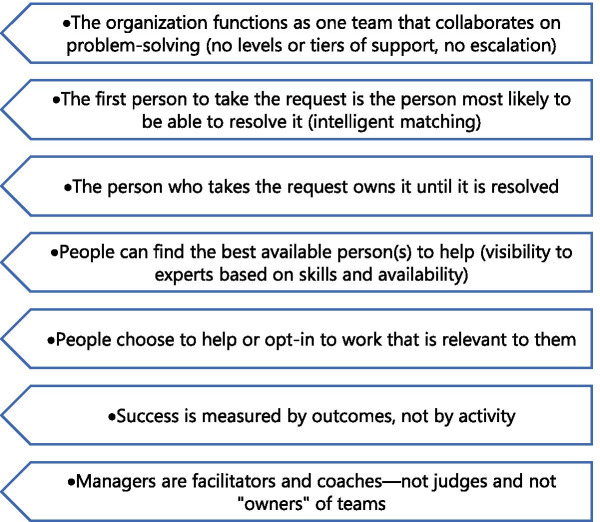
Principles of intelligent swarming

An idea for a dynamic support model, called intelligent swarming℠ [55], has been proposed in the technology industry as a way to provide more responsive, timely, and customized technical support. Drawing from the principles of agile software development, intelligent swarming replaces the traditional process of referring customers from generalist to experts with handoffs at each level, with a collaborative “swarm” of support personnel who best match the customer’s unique needs and are motivated and capable of providing the necessary assistance. For simple problems, the swarm could be a single person; for more complex problems, the customer is at the center of an interdisciplinary network of helpers that could include technical support staff, sales teams, strategic partners, or other customers. The approach is based on the principles listed in Fig. 2. It is instructive to speculate how such a model might work to meet the diverse learning needs of LMIC stakeholders. Figure 3 shows a network of support resources who could constitute a swarm.

One of the critical features of the swarm that makes it dynamic and adaptive is the matching process. This could be manual or automated, but the success of the swarm model will depend on the matchmaker’s ability to quickly assess, identify, and assemble the particular support team that meets the learning need. Depending on the need, this could be as simple as referral to relevant literature or instructional modules. For more complicated requests, such as the need to understand the use of a particular framework, the swarm may include networks of peers who have experience with the framework in various settings or consultation with local experts. For assistance with an implementation research proposal, the swarm might include implementation scientists, researchers, and program implementers with contextual knowledge about the setting. For policy makers seeking to use research data for decision-making, the swarm might include researchers, government personnel responsible for managing implementation, and frontline staff responsible for delivery. The learners themselves may be both customers of the swarm and suppliers, and willingness to contribute to the swarm might be imposed as a necessary precondition for access to resources. The swarm model may not always be a replacement for traditional training but may be a translational supplement to facilitate knowledge use. Initially, when local capacity in a particular setting is scarce, the networks from which swarms can be assembled might need to be global, and swarms must be carefully assembled to balance external expertise with local experience.

Intelligent swarming is still untested in these contexts and would need the infrastructure, incentives, and local capacity to make these models a reality. But we strongly believe that an emergent, adaptive approach is a powerful and innovative way to accommodate the enormous heterogeneity in background and skills among those involved in implementation-related activities in LMICs and to meet the enormous demand for capacity building in the field. We advocate for increased research efforts to develop and test swarming models for learning. As increasing numbers of local researchers and practitioners gain competency in key IS domains, learning networks with deeply rooted context-specific expertise can be developed, resulting in the availability of an equitable and appropriate body of implementation research knowledge closest to where it is most needed.

### Limitations

Although we employed a strategy meant to approximate a representative sample of each stakeholder group, misrepresentation could have skewed the results. Additionally, our positionality as researchers based in a HIC researching the learning needs of stakeholders in LMICs may have biased our methods and findings. However, we made several attempts to limit bias (e.g., member checking, critical moments rubric).

## Conclusions

This work is the first to explicitly explore and highlight the need for fundamental, widespread, and context-specific IS training and capacity building in conducting basic operational research for key stakeholders in LMICs. While many of the learning needs expressed by our interviewees are also issues in high-income settings, the scale of the gap between demand and existing capacity, the complex factors affecting access and availability, and the variation in expertise resulting from HIC initiated funding streams create a compelling case for innovative approaches that have not been tested before. We propose the novel approach of intelligent swarming as a solution to build IS capacity in LMICs through the lens of sustainability and equity.

## Supplementary Information

**Additional file 1.** Interview Guide.

**Additional file 2.** Critical Moments Rubric.

**Additional file 3.** Standards for Reporting Qualitative Research Checklist.

## Data Availability

The datasets used and/or analyzed during the current study are available from the corresponding author on reasonable request.

## Abbreviations

AHISA: Adolescent HIV Implementation Science Alliance
D&I: Dissemination and implementation
GACD: Global Alliance for Communicable Diseases
HIC: High-income countries
HIV: Human immunodeficiency virus
IS: Implementation science
LMIC: Low- and middle-income countries
NIH: National Institutes of Health
TB: Tuberculosis
