# Supplementary material for: Learning needs assessment for multi-stakeholder implementation science training in LMIC settings: findings and recommendations
Source: Implement Sci Commun. 2021 Dec 4;2:134. doi: 10.1186/s43058-021-00238-2 (PMC8642989; doi:10.1186/s43058-021-00238-2)
Supplement: Supplementary file 1 — Additional file 1. Interview Guide. [file 43058_2021_238_MOESM1_ESM.docx]

**Additional File 1: Interview Guide**

Country of work: _____________________________________

Profession/role: ____________________________

**Part A: Your experience with implementation science training**

1. How long have you been working in the area of implementation science?
2. How do you apply implementation science knowledge and tools in your work?
3. What implementation science topics and tools are most relevant to your work?
4. Possible probe: how would you define IS—do you use CFIR, other specific frameworks?
5. How did you learn about these topics? [please state all your training programs, including self study]
6. To what extent were your training programs useful in helping you learn what you need for your work?
7. What gaps were there between your training programs and what you need to know for your work? How did you close those gaps?
8. How do you continue to learn in this emerging field? How do you decide what new knowledge is most useful for you?

**Part B: Creating an optimal implementation science learning program**

1. Imagine someone in the same professional role as yourself who has not received any training in implementation science. What would an ideal training/support program that met your learning needs look like in terms of:

Probes:

1. Topics to be emphasized
2. Duration of training (single day short course, multiday course, multiple days spread over several months)
3. Delivery format (online, face to face, hybrid)
4. Training mode (self study with expert support, didactic teaching of key concepts, workshop mode with case study/worked examples)
5. Training participants (people with similar roles, people with different roles in the same training program)
6. Training instructors (what kinds of peoples should teach, and how should they collaborate?)
7. Post-training support/opportunities for ongoing learning?

10. Do you have any other thoughts about the best way to enhance implementation science learning for someone in your role?
