# Supplementary material for: Learning needs assessment for multi-stakeholder implementation science training in LMIC settings: findings and recommendations
Source: Implement Sci Commun. 2021 Dec 4;2:134. doi: 10.1186/s43058-021-00238-2 (PMC8642989; doi:10.1186/s43058-021-00238-2)
Supplement: Supplementary file 2 — Additional file 2. Critical Moments Rubric. [file 43058_2021_238_MOESM2_ESM.docx]

**Additional File 2. Critical Moments Rubric**

RUBRIC FOR DOUBLE LOOP LEARNING ASSESSMENT OF THE LEARNING NEEDS INTERVIEW ANALYSIS

Double Loop Learning Example:

*To give a simple analogy: a thermostat that automatically turns on the heat whenever the temperature in a room drops below 68 degrees is a good example of single-loop learning. A thermostat that could ask, “Why am I set at 68 degrees?” and then explore whether or not some other temperature might more economically achieve the goal of heating the room would be engaging in double-loop learning*(Argyris, 1977*).*

Basis for the rubric:

*Derived from the frameworks on Critical Moments by McDowell et al. (2005) and on Reflection on Action by Smyth (1989) as reported by Greenwood (1998).*

- As you reflect on this interview, were there “critical moments” that stood out you as representative of the LMIC context?
- Why do you consider these to be critical moments?

**MEMO LEVEL RUBRIC**

| **Interview #** | - **As you reflect on this interview, were there “critical moments” that stood out you as representative of the LMIC context?** - **Why do you consider these to be critical moments?** |
| --- | --- |
