# Supplementary material for: Learning needs assessment for multi-stakeholder implementation science training in LMIC settings: findings and recommendations
Source: Implement Sci Commun. 2021 Dec 4;2:134. doi: 10.1186/s43058-021-00238-2 (PMC8642989; doi:10.1186/s43058-021-00238-2)
Supplement: Supplementary file 3 — Additional file 3. Standards for Reporting Qualitative Research Checklist. [file 43058_2021_238_MOESM3_ESM.doc]

**Standards for Reporting Qualitative Research (SRQR)**

O’Brien B.C., Harris, I.B., Beckman, T.J., Reed, D.A., & Cook, D.A. (2014). Standards for reporting qualitative research: a synthesis of recommendations. *Academic Medicine, 89(9)*, 1245-1251.

| **No. Topic** | **Page Number** |
| --- | --- |
| **Title and abstract** |  |
| S1 Title | 1 |
| S2 Abstract | 2 |
| **Introduction** |  |
| S3 Problem formulation | 5 |
| S4 Purpose or research question | 6 |
| **Methods** |  |
| S5 Qualitative approach and research paradigm | 9 |
| S6 Researcher characteristics and reflexivity | 10-11 |
| S7 Context | 5 |
| S8 Sampling strategy | 8 |
| S9 Ethical issues pertaining to human subjects | 27 |
| S10 Data collection methods | 9 |
| S11 Data collection instruments and technologies | Supplementary Files 1 and 2 |
| S12 Units of study | 12 |
| S13 Data processing |  |
| S14 Data analysis | 11-12 |
| S15 Techniques to enhance trustworthiness | 9-10 |
| **Results/Findings** |  |
| S16 Synthesis and interpretation | 12-19 |
| S17 Links to empirical data | 12-19 |
| **Discussion** |  |
| S18 Integration with prior work, implications, transferability, and contribution(s) to the field | 20-23 |
| S19 Limitations | 24 |
| **Other** |  |
| S20 Conflicts of interest | 27 |
| S21 Funding | 27 |

.
